# Combination of Mussel Inspired Method and “Thiol-Michael” Click Reaction for Biocompatible Alginate-Modified Carbon Nanotubes

**DOI:** 10.3390/nano11092191

**Published:** 2021-08-26

**Authors:** Haiyan Yao, Mingzhi Zhu, Pei Wang, Yuangang Liu, Junchao Wei

**Affiliations:** 1 School of Stomatology, Nanchang University, Nanchang 330006, China; ncuyaohaiyan@163.com (H.Y.); ndfskqyy620@ncu.edu.cn (P.W.); 2 College of Chemistry, Nanchang University, Nanchang 330031, China; 3 The Key Laboratory of Oral Biomedicine, Nanchang 330006, China; 4 College of Chemical Engineering, Huaqiao University, Xiamen 361021, China; 15762217960@163.com (M.Z.); ygliu@hqu.edu.cn (Y.L.)

**Keywords:** carbon nanotube, alginate, mussel-inspired, click reaction, surface modification

## Abstract

Carbon nanotubes (CNTs) have attracted great interest in biomedical fields. However, the potential toxicity and poor dispersion of CNTs have greatly limited its application. In this work, a mussel-inspired method combined with the “thiol-Michael” click reaction was used to modify the surface of CNT and improve its properties. Firstly, a CNT was treated with dopamine, and then alginate grafted with L-cysteine was anchored onto the surface of CNT via click reaction, which realized the long-time dispersion of CNT in water. Furthermore, the in vitro test also demonstrated that the alginate may improve the biocompatibility of CNT, and thus may broaden the application of CNT in the biomedical field.

## 1. Introduction

Due to its amazing mechanical, electrical and thermal properties, carbon nanotubes (CNT) and other carbon-based materials have been widely used in composite materials [1], energy conversion and storage services [2], catalysts [3], sensors [4,5], drug delivery [6,7], antiviral agents [8], tissue engineering [9] and many promising fields [10,11,12]. However, because of the high surface energy and strong π-π interactions, CNT are prone to aggregate heavily, which makes it difficult to disperse CNT in various liquids or matrix [13,14]; furthermore, the aggregation of CNT also increases its toxicity and hinder its application in biomedical related fields [15,16]. Thus, it is very critical to hinder the aggregation of CNT to improve its application.

Up to now, various methods, both non-covalent and covalent, have been widely investigated to tune the surface properties of CNT and realize their homogeneous dispersion [17]. The noncovalent approaches are easily carried out and low cost. Various molecules, such as aromatic small molecules, polysaccharides, conjugated polymers, have been used to modify the surface of CNT through noncovalent π-π stacking or hydrophobic interactions [18]. The noncovalent method can improve the dispersion state or solubility of CNT and offers excellent properties to be used in various nanomaterials and devices. However, one drawback for the noncovalent method is its stability, which means that the molecules adhered to the surface of CNT may be detached from the CNT under certain conditions. On the contrary, the covalent approaches to functional CNT mainly rely on chemical reactions between special functional molecules with the functional groups on CNT [19]. For example, the defect sites of CNT can be oxidized into carboxyl groups, which can further react with other molecules or even nanoparticles via esterification or amination reactions. Moreover, some special initiators may be anchored on the surface of CNT via covalent reaction, and then be used to initiate the polymerization of some monomers and formed polymer brushes on the surface of CNT. Many classical methods, such as atom transfer radical polymerization (ATRP) [20], reversible addition-fragmentation chain transfer (RAFT) [21] and ring-opening polymerization (ROP) [22] have been well used to create various functionalized CNT. For example, amphiphilic polymer brush-modified CNT, displaying good dispersibility both in chloroform and water, have been built by uniting click chemistry and ATRP [23]. However, these methods require either severe reaction conditions or complex preparation methods; furthermore, toxicity catalysts are always used. Thus, it is still very important to develop simple and low-cost methods to preparation stable and biocompatible CNT to promote their application.

Inspired by the composition of adhesive proteins in mussels, dopamine was used as a monomer, and it can be self-polymerized into polydopamine (PDA) film on the surface of various materials easily. Furthermore, the PDA film contains abundant functional groups and can react with many other molecules or particles, and thus this method has been widely called the “mussel inspired” method and has been used to realize the surface functionalization of various materials [24,25]. For example, gelatin molecules were grafted on the surface of PDA-modified carbon nanotube via Michael addition reaction between the amino groups of gelatin and the quinone units of PDA [26]. More interestingly, the “thiol-Michael” click reaction is also a widely used mild method to prepare various functional materials [27,28,29]. Thus, due to the high activity of the quinone units of PDA, many other functional molecules may be attached to the surface of PDA-coated materials via the “thiol-Michael” click reaction.

**Figure 1:**
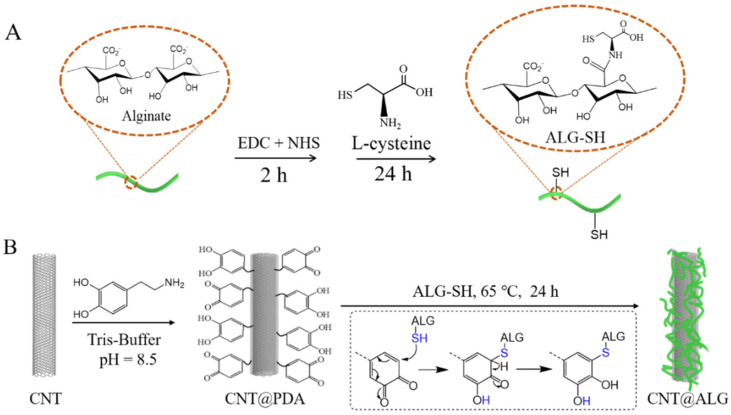
Schematic illustration of the construction of (**A**) ALG-SH and (**B**) CNT@ALG.

In this work, we try to propose a simple and mild method to modify the surface of CNT by biocompatible polymer, and we selected alginate (ALG) as a biocompatible model. Firstly, ALG was activated with EDC/NHS, and then reacted with L-cysteine to prepare thiol groups functionalized alginate molecules (ALG-SH) (Figure 1). Secondly, CNT was treated with dopamine in Tris-buffer solution (pH = 8.5) and, thus, PDA-coated CNT (CNT@PDA) were prepared via the mussel-inspired method, which formed dihydroxylphenol and quinone fragments on the surface of CNT [24]. Then, alginate-modified CNT (CNT@ALG) were obtained via the click reaction between ALG-SH and PDA on the surface of CNT. The dispersion and biocompatibility test showed that CNT@ALG can maintain good dispersion in water for a long time, even up to one month. What is more, the biocompatibility is also better than that of native CNT, implying that the modified CNT would have great potential applications in biomedical fields and nanocomposites.

## 2. Materials and Methods

### 2.1. Materials

CNT was purchased from Xianfeng Nanomaterial Technology Co., Ltd. (Nanjing, China). Dopamine hydrochloride (98%), ethyl-3-(3-dimethylaminopropyl) carbodiimide hydrochloride (EDC), N-hydroxysuccinimide (NHS, 98%), sodium alginate (98%), and tris (hydroxymethyl) aminomethane hydrochloride (98%) were purchased from Aladdin Scientific Co., Ltd. (Shanghai, China). Tris (hydroxymethyl) aminomethane (98%), cysteine (98%) was purchased from Sinopharm Chemical Reagent Co., Ltd. (Shanghai, China). Acridine orange/ethidium bromide was purchased from Solarbio Co., Ltd. (Beijing, China). Cell Counting Kit-8 (CCK-8) was purchased from KeyGene Biotech Co., Ltd. (Nanjing, China).

### 2.2. Methods

#### 2.2.1. Preparation of Dopamine Coated CNT (CNT@PDA)

CNT (250 mg) was dispersed in 125 mL tris-buffer (pH = 8.5) via ultrasonication, and then 250 mg dopamine was added into the dispersion system under stirring. Then, 24 h later, the product (CNT@PDA) was collected via centrifugation, and washed with deionized water and lyophilized.

#### 2.2.2. Preparation of Alginate-Modified CNT (CNT@ALG)

Firstly, ALG was functionalized with L-cysteine (ALG-SH) to introduce thiol groups on the chains of ALG via carbodiimide-mediated chemistry [30]. Briefly, alginate (1 g) was added into 100 mL deionized water and stirred for 12 h, and then 0.9585 g EDC and 0.5777 g NHS was added into the alginate solution and stirred for 2 h to activate the carboxyl group. Then, L-cysteine (0.5 g) was added to the above solution and stirred for 24 h. Finally, ALG-SH was purified via dialysis in deionized water.

Secondly, ALG-modified CNT (CNT@ALG) was obtained via click reaction between CNT@PDA and ALG-SH. Briefly, the CNT@PDA was added into the above ALG-SH solution and stirred for 24 h at 65 °C. The alginate-modified CNT (CNT@ALG) were collected by centrifuging and then were washed with deionized water and lyophilized.

#### 2.2.3. Characterization

The successful grafting of ALG on CNT was demonstrated by Fourier transform infrared spectroscopy (FTIR, Agilent Technologies, Cary6302400304, Santa Clara, CA, USA). The elements of various samples were determined by an X-ray photoelectron spectrometer (XPS, Thermo Fisher Scientific, ESCALAB 250, Waltham, MA, USA). The weight loss of different samples was measured by a thermal gravimetric analyzer (TGA, PerkinElmer, TGA 400003030247, Waltham, MA, USA). The morphology of different samples was observed by scanning electron microscopy (SEM, Phenom World B.V, Phenom LE, Eindhoven, The Netherlands).

#### 2.2.4. Dispersion Test

Dispersion of different samples was observed by dispersing them in water (5 mg/mL) and taking photos at different time intervals.

#### 2.2.5. Biocompatibility Test

The biocompatibility of various samples was assessed via the CCK-8 method and acridine orange/ethidium bromide (AO/EB) staining. Firstly, the CCK-8 method was used to calculate the cell viability of the MC3T3-E1 cell line and HepG2 cell line. An amount of 100 μL DMEM with 10% (*v*/*v*) FBS containing cells at a density of 8 × 10^4^ cells/mL was seeded into a 96-well plate and incubated at 37 °C under an atmosphere of 5% CO_2_ for 24 h. Subsequently, the culture media was replaced with 100 μL of fresh medium containing materials at various concentrations of 5, 10, 25, 50 µg/mL, respectively. After the cells were co-incubated with samples for 24 h, the culture media was removed and then the wells were washed with fresh medium several times, and CCK-8 reagent (10 μL) mixed with the 100 μL of fresh medium was added to each well. After 2 h of incubation, the absorbance of the solution was measured by using a spectrophotometer (Thermo Scientific, Varioskan Flash 1510, Waltham, MA, USA) at a wavelength maximum of 450 nm. Cell viability was calculated by using the following formula:

(1) $$\mathrm{Cell}\;\mathrm{viability}=\frac{{\mathrm{OD}}_{\mathrm{treated}}\;-\;{\mathrm{OD}}_{\mathrm{free}}}{{\mathrm{OD}}_{\mathrm{control}}\;-\;{\mathrm{OD}}_{\mathrm{free}}}\;\times \;100\%$$

where OD_treated_ is the absorbance of the group with samples, OD_free_ is the absorbance of the group of without cells in well, OD_control_ is the control group of cells cultured without sample treatment. Each sample was repeated 5 times and the results were shown as mean ± SD (standard deviation). Moreover, the significant difference between samples was analyzed via Student’s test with Graphpad Prism 8.

AO/EB staining was used to detect the apoptosis of MC3T3-E1 cells. An amount of 1 mL DMEM with 10% (*v*/*v*) FBS containing MC3T3-E1 cells at a density of 8 × 10^4^ cells/mL was seeded into a 24-well plate (containing the dish climbing glasses) and incubated at 37 °C under an atmosphere of 5% CO_2_ for 24 h. Subsequently, the culture media was replaced with 1 mL of fresh medium containing samples at various concentrations of 5, 10, 25, 50 µg/mL. After 24 h co-incubation, fresh medium was used to replace the old medium to wash each well several times, and then AO/EB reagent was added to each well for 30 s. Finally, the survival of cells was observed by Fluorescence microscopy.

## 3. Results and Discussion

### 3.1. Characterization of Different Samples

Dopamine tends to self-polymerize under an alkaline environment. When CNT and dopamine are mixed together in a buffer solution with a pH value of 8.5, dopamine will self-polymerize, and PDA layers will be formed on the surface of CNT. The PDA molecule contains a quinone structure, and can react with thiol groups (–SH) under mild conditions easily and efficiently [24,31], which was dubbed a kind of click-chemistry [32]. Therefore, when ALG-SH was mixed with CNT@PDA, the quinone units will react quickly with thiol groups of ALG-SH, and thus, ALG molecules were successfully grafted on the surface of CNT, forming CNT@ALG (Figure 1).

**Figure 2:**
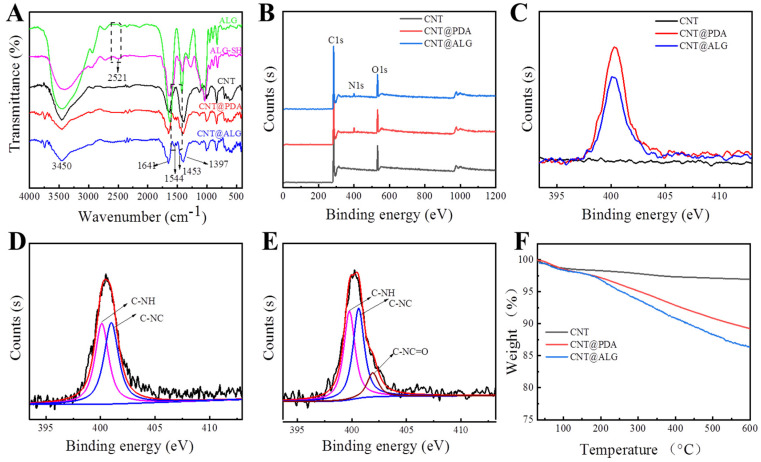
(**A**) FTIR spectra of ALG, ALGSH, CNT, CNT@PDA, CNT@ALG; (**B**) XPS spectra of CNT, CNT@PDA, CNT@ALG; (**C**) XPS spectra of N1s of CNT, CNT@PDA, CNT@ALG; (**D**) High-resolution XPS spectra of N1s of CNT@PDA; (**E**) High-resolution XPS spectra of N 1s of CNT@ALG; (**F**) TGA curves.

FT-IR measurements were employed to demonstrate the successful grafting of alginate on CNT (Figure 2A). In the spectra of ALG, a wide absorption peak appeared at 3450 cm^−1^, as well as in the spectra of ALG-SH. Meanwhile, a peak related to –SH appeared at 2521 cm^−1^ [33], which demonstrated that L-cysteine-grafted alginate (ALG-SH) was successfully prepared. Wide absorption bands around 3450 cm^−1^, 1641 cm^−1^, 1397 cm^−1^ in the spectra of CNT were attributed to the stretching vibration of –OH, C=O and the bending vibration of –OH from the carboxyl group in CNT [34], respectively. However, new absorption peaks appeared at 1544 cm^−1^ and 1453 cm^−1^ in the spectra of CNT@PDA and CNT@ALG, which were attributed to the bending vibration of N–H and the bending vibration of –CH_2_–; furthermore, the weak peak at 2521 cm^−1^ decreased in the spectrum of CNT@ALG, meaning that the –SH group did not exist in CNT@ALG, implying that the –SH group reacted with the quinone group and formed C–S covalent bonds. Thus, these results represented the successful polymerization of dopamine on the surface of CNT and the successful grafting of ALG.

XPS measurements were carried out to investigate the surface composition of CNTs and the modified CNT. The characteristic peak of C1s in CNT was 284.58 eV and O1s was 532.8 eV, respectively (Figure 2B). In the XPS spectra of CNT@PDA, the peak at 400.2 eV was the signal of N1s, which proved that PDA was successfully coated on the surface of CNT. Furthermore, the quantitative XPS results showed that the N atom ratio on the surface of CNT@PDA was about 3.46%. As for CNT@ALG, the signal peak of N1s also appeared (Figure 2B,C); however, the N atom ratio was only 2.93%, much lower than that of CNT@PDA, due to the nitrogen content of ALG-SH being smaller than that of PDA, and thus, when ALG-SH was connected on the surface of CNT@PDA, the nitrogen content was relatively reduced. In the N1s spectrum of CNT@PDA, two peaks could be fitted, which are assigned to the C–N–H (400.2 eV) on the amino group and the C–N–C (401.1 eV) in the indole structure (Figure 2D) from PDA [35]. Meanwhile, there are three peaks in N1s of CNT@ALG, which are attributed to the C–N–H (399.8 eV) on the amino group, the C–N–C (400.6 eV) in the indole structure and the C–N–C=O from the amide in ALG-SH [36] (Figure 2E). These results also demonstrated that dopamine was successfully polymerized on the CNT surface and ALG was grafted onto CNT@PDA successfully.

In order to evaluate the grafting amount of alginate, TGA was used to test the weight loss of different samples (Figure 2F). The TGA results showed that the weight loss of CNT was 1.6% when heated to 150 °C, which may be attributed to the evaporation of the absorbed solvent and the 1.4% weight loss between 150 °C to 600 °C was due to the decomposition of carboxyl groups of CNT. Moreover, the removal of amorphous carbon can also result in weight loss in this range. The weight loss of CNT@PDA was 10.9% when the temperature rose from 30 °C to 600 °C, which could be defined in two steps: the 2.1% evaporation of absorbed solvent before 150 °C and 8.8% decomposition of PDA between 150 °C to 600 °C, proving that the PDA was successfully coated on the surface of CNT. As for CNT@ALG, the weight loss of CNT@ALG before 150 °C and 150 °C to 600 °C was 2.1% and 11.5%, attributed to the evaporation of solvent and decomposition of PDA and ALG, respectively (Figure 2F). These results further demonstrate the successful polymerization of dopamine on the surface of CNT and the successful grafting of alginate.

### 3.2. Dispersion Test

**Figure 3:**
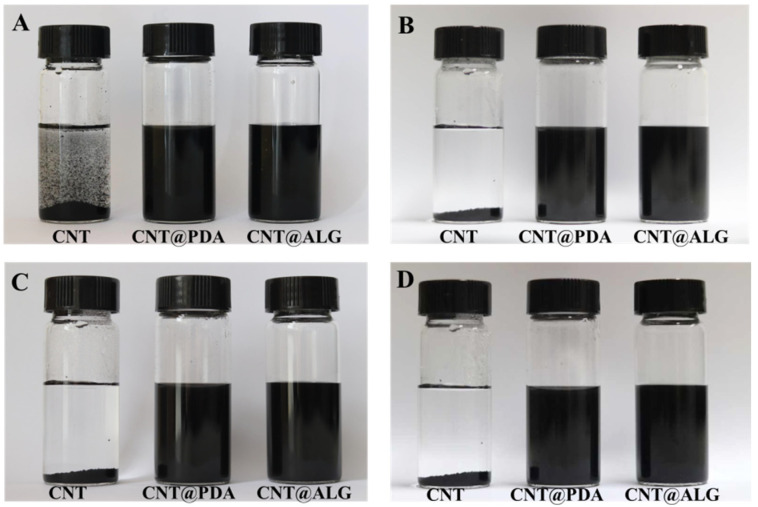
The photos of CNT, CNT@PDA, CNT@ALG after dispersed in water for 1 min (**A**), 4 h (**B**), 24 h (**C**) and 1 week (**D**).

Due to the strong π–π interactions, CNT are prone to aggregate heavily, and it is difficult to be dispersed in a liquid solution or polymer matrix. When CNT are dispersed in water under ultrasonication, they are unable to form a stable dispersion system. Even after one minute, the CNT would precipitate rapidly to the bottom of the bottle (Figure 3A). Instead, when PDA was coated on the surface of CNT, the PDA molecules may prevent the aggregation of CNT, and thus, the CNT@PDA could disperse well in water (Figure 3A). Furthermore, ALG is a kind of hydrophilic polymer, and when grafted on the surface of CNT, it may change the surface properties of CNT from hydrophobic to hydrophilic, and thus the CNT@ALG can disperse homogeneously in water (Figure 3A). As shown in Figure 3B–D, the CNT@PDA and CNT@ALG showed much better colloid stability, as even after one week, the CNT@PDA and CNT@ALG could still disperse very well (Figure 3D). As a matter of fact, after one month, the CNT@ALG still dispersed very well, demonstrating that the ALG molecules on the surface of CNT can work as polymer brushes to prevent the aggregation of CNT.

**Figure 4:**
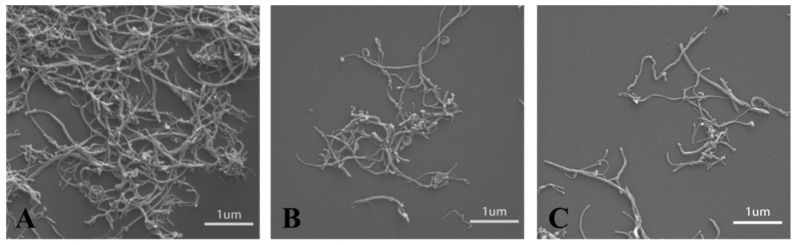
SEM images of CNT (**A**), CNT@PDA (**B**) and CNT@ALG (**C**).

CNT have great potential applications as nanofillers in polymer composites. However, the mechanical properties of the reinforced polymer composites are always far below their expected theoretical values. The deficiency in the properties of polymer composites is related to the difficulty of obtaining the good dispersion of nanofillers. An efficient way is to modify the nanofillers with polymers and increase the phase compatibility between nanofillers and polymers. Herein, the CNT can entangle in bundles even at very low concentrations (Figure 4A), which is a great drawback for its application in polymer composites. However, due to the presence of PDA or ALG on the surface of CNT, the hybrid CNT@PDA and CNT@ALG can disperse well (Figure 4B,C). These results were in accordance with the dispersion test, demonstrating that the grafting of ALG on the surface of CNT may greatly change its surface properties. This change may tune the phase interaction between CNT and other polymer matrices, and thus may make CNT@ALG a novel filler to reinforce polymers and improve their mechanical properties. Generally, it has been a common method to graft hydrophilic polymers, such as PEG-based polymers [37] and gelatin [26], on the surface of CNT to improve its dispersion state. Thus, we are sure that the prepared CNT@ALG has great potential in polymer composites and realizes the improvement of mechanical properties, which may broaden the application of CNT in reinforced nanocomposites.

### 3.3. Biocompatibility Test

**Figure 5:**
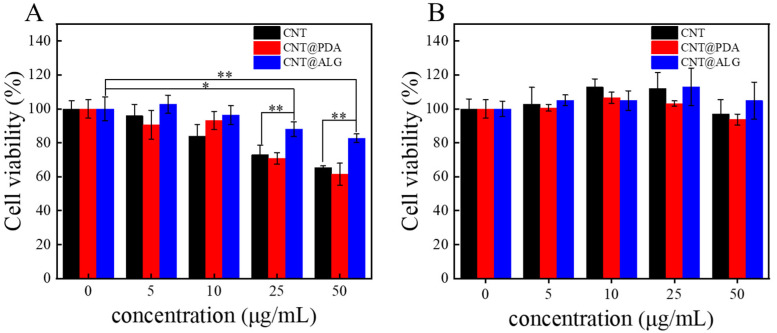
Cell viability of MC3T3-E1 cells (**A**) and HepG2 cells (**B**) cultured in suspensions of CNT, CNT@PDA, CNT@ALG at different concentrations. (*n* = 5), * *p* < 0.05, ** *p* < 0.01.

Although many works have reported that CNT are biocompatible and can be well used in biomedical fields, such as biosensing, diagnosis and cancer therapy, it is still of great importance to tune the surface properties of CNT and improve its biocompatibility. Since ALG is a biocompatible polymer, when it was grafted on the surface of CNT, the dispersion state of CNT was greatly changed. Furthermore, it may improve the interactions between the CNT and cells, and thus, the biocompatibility of CNT may be improved. As shown in Figure 5A, when the concentration of different samples was 5 μg/mL and 10 μg/mL, CNT, CNT@PDA and CNT@ALG all showed great biocompatibility to MC3T3-E1 cells, with the increase in sample concentration, the cell viability of CNT and CNT@PDA decreased a lot, however, the results of CNT@ALG was much better. Even at a concentration of 50 μg/mL, the cell viability of CNT@ALG was 83 ± 2.5%, much higher than that of CNT (65 ± 1.14%) and CNT@PDA (61 ± 6.4%) (Figure 5A). These results may imply that the toxicity of CNT was concentration-dependent, which has a similar tendency as that of PEGylated graphene nanoribbons [38]; however, the threshold concentration was different. When HepG2 cells were used, all the samples showed good biocompatibility; however, the CNT@ALG still showed slightly better results than CNT. For example, when the sample concentration was 50 μg/mL, the cell viability of CNT@ALG was about 105.4 ± 10.9%, while the results of CNT and CNT@PDA were 97 ± 8.5% and 93 ± 3.2%, respectively (Figure 5B). When compared with Figure 5A,B, it was found that the cell viability between different types of cells was slightly different. We think that this is a general phenomenon, because the surface composition of different cells may have minor differences, which may affect the interactions between cells and nanomaterials. However, both the results in Figure 5A,B may confirm that the CNT@ALG is biocompatible, implying its potential biomedical applications.

**Figure 6:**
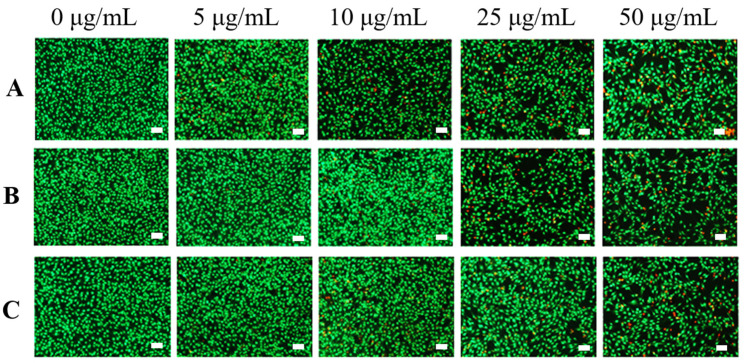
AO/EB staining assay of MC3T3-E1 cells cultured with various samples CNT (**A**), CNT@PDA (**B**), CNT@ALG (**C**) in different concentration. (100× magnification, Bar = 100 μm).

AO/EB staining was carried out to further evaluate the biocompatibility of various samples. Figure 6 showed that most of the cells survived (green fluorescence) when the samples were at low concentration (5 μg/mL), proving that the samples had low biotoxicity in this concentration. When the concentration increased to 25 μg/mL, the number of dead cells (red fluorescence) in CNT and CNT@PDA was slightly increased, while there were still few dead cells in the CNT@ALG group. When the concentration reached 50 μg/mL, there were fewer dead cells in CNT@ALG compared with that of CNT and CNT@ALG, demonstrating that CNT@ALG had lower cytotoxicity even at high concentrations. These results indicated that the modification of CNT with biocompatible ALG could significantly enhance the biocompatibility of CNT. Furthermore, due to the numerous merits, including biocompatibility and ease of gelation, ALG has been attractive in wound dressing, drug delivery, and tissue engineering. The ALG@CNT prepared in this work can combine the advantages of CNT and ALG, and may be blended with ALG to prepare various types of functional materials to be applied in the biological field, such as biosensors, tissue engineering and electrical implants.

## 4. Conclusions

In this work, alginate was grafted onto the surface of CNT via the combination of the mussel-inspired method and “thiol-Michael” click chemistry. The results showed that the alginate-modified CNT (CNT@ALG) can disperse well in water for a long time, which may be very helpful for the application of carbon nanotubes as nanofillers in nanocomposites. Moreover, the CNT@ALG showed better biocompatibility than native CNT, demonstrating that grafting biocompatible polymers on the surface of CNT is a method to decrease its toxicity, which provides more possibilities for the application of CNT in the biological field.

## Data Availability

The data presented in this study are available on request from the corresponding author.

## References

[1] Coleman J.N., Khan U., Blau W.J., Gun’ko Y.K. (2006). Small but strong: A review of the mechanical properties of carbon nanotube–polymer composites. Carbon.

[2] Kumar S., Nehra M., Kedia D., Dilbaghi N., Tankeshwar K., Kim K.-H. (2018). Carbon nanotubes: A potential material for energy conversion and storage. Prog. Energy Combust. Sci..

[3] Yan Y., Miao J., Yang Z., Xiao F.X., Yang H.B., Liu B., Yang Y. (2015). Carbon nanotube catalysts: Recent advances in synthesis, characterization and applications. Chem. Soc. Rev..

[4] Schroeder V., Savagatrup S., He M., Lin S., Swager T.M. (2019). Carbon Nanotube Chemical Sensors. Chem. Rev..

[5] Muhulet A., Miculescu F., Voicu S., Schütt F., Thakur V., Mishra Y. (2018). Fundamentals and scopes of doped carbon nanotubes towards energy and biosensing applications. Mater. Today Energy.

[6] Bianco A., Kostarelos K., Prato M. (2005). Applications of carbon nanotubes in drug delivery. Curr. Opin. Chem. Biol..

[7] Zhang J., Liu Z., Zhou S., Teng Y., Zhang X., Li J. (2020). Novel Span-PEG Multifunctional Ultrasound Contrast Agent Based on CNTs as a Magnetic Targeting Factor and a Drug Carrier. ACS Omega.

[8] Serrano-Aroca A., Takayama K., Tunñón-Molina A., Seyran M., Hassan S., Choudhury P.N., Uversky V., Lundstrom K., Adadi P., Palù G. (2021). Carbon-based nanomaterials: Promising antiviral agents to combat COVID-19 in the microbial-resistant era. ACS Nano.

[9] Li Y., Qin S., Peng J., Chen A., Nie Y., Liu T., Song K. (2020). Engineering gelatin-based alginate/carbon nanotubes blend bioink for direct 3D printing of vessel constructs. Int. J. Biol. Macromol..

[10] Akhavan O., Ghaderi E., Shahsavar M. (2013). Graphene nanogrids for selective and fast osteogenic differentiation of human mesenchymal stem cells. Carbon.

[11] Akhavan O., Ghaderi E. (2013). Differentiation of human neural stem cells into neural networks on graphene nanogrids. J. Mater. Chem. B.

[12] De Volder M.F.L., Tawfick S.H., Baughman R.H., Hart A.J. (2013). Carbon Nanotubes: Present and Future Commercial Applications. Science.

[13] Xie X., Mai Y., Zhou X. (2005). Dispersion and alignment of carbon nanotubes in polymer matrix: A review. Mater. Sci.Eng. R Rep..

[14] Hao M., Tang M., Wang W., Tian M., Zhang L., Lu Y. (2016). Silver-nanoparticle-decorated multiwalled carbon nanotubes prepared by poly(dopamine) functionalization and ultraviolet irradiation. Compos. Part B Eng..

[15] Alshehri R., Ilyas A.M., Hasan A., Arnaout A., Ahmed F., Memic A. (2016). Carbon Nanotubes in Biomedical Applications: Factors, Mechanisms, and Remedies of Toxicity. J. Med. Chem..

[16] Liu Y., Zhao Y., Sun B., Chen C. (2013). Understanding the toxicity of carbon nanotubes. Acc. Chem. Res..

[17] Sahoo N.G., Rana S., Cho J.W., Li L., Chan S.H. (2010). Polymer nanocomposites based on functionalized carbon nanotubes. Prog. Polym. Sci..

[18] Zhao Y., Stoddarts J.J. (2009). Noncovalent functionalization of single-walled carbon nanotubes. Acc. Chem. Res..

[19] Nikolaos K., Nikos T. (2010). Current progress on the chemical modification of carbon nanotubes. Chem. Rev..

[20] Huang H., Liu M., Xu D., Mao L., Huang Q., Deng F., Tian J., Wen Y., Zhang X., Wei Y. (2020). Facile fabrication of glycosylated and PEGylated carbon nanotubes through the combination of mussel inspired chemistry and surface-initiated ATRP. Mater. Sci. Eng. C Mater. Biol. Appl..

[21] Shi Y., Zeng G., Xu D., Liu M., Wang K., Li Z., Fu L., Zhang Q., Zhang X., Wei Y. (2017). Biomimetic PEGylation of carbon nanotubes through surface-initiated RAFT polymerization. Mater. Sci. Eng. C.

[22] Li J., Zhao L., Wang W., Liu Y., Yang H., Kong J., Si F. (2021). Polymer-functionalized carbon nanotubes prepared via ring-opening polymerization for electrochemical detection of carcinoembryonic antigen. Sens. Actuators B Chem..

[23] Zhang Y., He H., Gao C. (2008). Clickable macroinitiator strategy to build amphiphilic polymer brushes on carbon nanotubes. Macromolecules.

[24] Lee H., Dellatore S.M., Miller W.M., Messersmith P.B. (2007). Mussel-inspired surface chemistry for multifunctional coatings. Science.

[25] Han L., Liu K., Wang M., Wang K., Fang L., Chen H., Zhou J., Lu X. (2018). Mussel-inspired adhesive and conductive hydrogel with long-lasting moisture and extreme temperature tolerance. Adv. Funct. Mater..

[26] Li D., Li S., Liu J., Zhan L., Wang P., Zhu H., Wei J. (2020). Surface modification of carbon nanotube with gelatin via mussel inspired method. Mater. Sci. Eng. C Mater. Biol. Appl..

[27] Escorihuela J., Marcelis A.T.M., Zuilhof H. (2015). Metal-Free Click Chemistry Reactions on Surfaces. Adv. Mater. Interfaces.

[28] Gennari A., Wedgwood J., Lallana E., Francini N., Tirelli N. (2020). Thiol-based michael-type addition. A systematic evaluation of its controlling factors. Tetrahedron.

[29] Pupkaite J., Rosenquist J., Hilborn J., Samanta A. (2019). Injectable Shape-Holding Collagen Hydrogel for Cell Encapsulation and Delivery Cross-linked Using Thiol-Michael Addition Click Reaction. Biomacromolecules.

[30] Yan N., Wang X., Lin L., Song T., Sun P., Tian H., Liang H., Chen X. (2018). Gold nanorods electrostatically binding nucleic acid probe for in vivo microRNA amplified detection and photoacoustic imaging-guided photothermal therapy. Adv. Funct. Mater..

[31] Krger J.M., Bçrner G.H. (2021). Accessing the next generation of synthetic Mussel-Glue polymers via mussel-inspired polymerization. Angew. Chem. Int. Ed..

[32] Nair D.P., Podgorski M., Chatani S., Gong T., Xi W., Fenoli R.C., Bowman C.N. (2014). The thiol-michael addition click reaction: A powerful and widely used tool in materials chemistry. Chem. Mater..

[33] Li J., Chen R., Zhang S., Ma Z., Luo Z., Gao G. (2019). Chiral Effect at Nano-Bio Interface: A Model of Chiral Gold Nanoparticle on Amylin Fibrillation. Nanomaterials.

[34] Shuai C., Zan J., Deng F., Yang Y., Peng S., Zhao Z. (2021). Core–SHell-Structured ZIF-8@PDA-HA with Controllable Zinc Ion Release and Superior Bioactivity for Improving a Poly-l-lactic Acid Scaffold. ACS Sustain. Chem. Eng..

[35] Li W., Li Y., Sheng M., Cui S., Wang Z., Zhang X., Yang C., Yu Z., Zhang Y., Tian S. (2019). Enhanced Adhesion of Carbon Nanotubes by Dopamine Modification. Langmuir ACS J. Surf. Colloids.

[36] Wu X., Tang Z., Liao X., Wang Z., Liu H. (2020). Fabrication of chitosan@calcium alginate microspheres with porous core and compact shell, and application as a quick traumatic hemostat. Carbohydr. Polym..

[37] Ravelli D., Montanaro S., Tomasi C., Galinetto P., Quartarone E., Merli D., Mustarelli P., Fagnoni M. (2012). One-Step Decatungstate-Photomediated PEGylation of Single-Walled Carbon Nanotubes. ChemPlusChem.

[38] Akhavan O., Ghaderia E., Emamy H. (2012). Nontoxic concentrations of PEGylated graphene nanoribbons for selective cancer cell imaging and photothermal therapy. J. Mater. Chem..

